# Effect of preoperative clear carbohydrate beverage on emergence delirium in children – a randomized controlled trial^☆^

**DOI:** 10.1016/j.bjane.2025.844698

**Published:** 2025-11-03

**Authors:** Karan Cheema, Aakriti Gupta, Ajay Singh, Preethy J. Mathew

**Affiliations:** a All India Institute of Medical Sciences, Department of Anaesthesiology and Critical Care, Jodhpur, India; b Postgraduate Institute of Medical Education and Research (PGIMER), Department of Anaesthesia and Intensive Care, Chandigarh, India

*Dear Editor,*

Preoperative carbohydrate loading has been shown to attenuate insulin resistance, maintain euglycemia and improve recovery in pediatric patients.^1^ Children are particularly vulnerable to perioperative glucose fluctuations due to limited metabolic reserves. Both hypoglycemia and hyperglycemia have been linked to cognitive dysfunction and delirium.^2^^,^^3^ Despite these associations, the effect of carbohydrate loading on Emergence Delirium (ED) remains underexplored. This study investigates whether preoperative carbohydrate drink administration reduces ED in children, focusing on fasting duration and blood glucose levels as potential mediators.

This prospective, randomized, assessor-blinded study included ASA I–II children aged 2–6 years scheduled for elective ocular surgery. Standard fasting guidelines were followed (> 6h for solids and > 2h for clear liquids). Participants were randomly allocated into:

• Group CD (Carbohydrate Drink): Received 3 mL.kg^-1^ (max 150 mL) of a commercially available clear carbohydrate beverage 2h preoperatively.
• Group C (Control): Received an equivalent volume of plain water at the same time interval.

A nurse uninvolved in the study administered the intervention. The same commercial brand of a flavored sweetened beverage (glucose content 12.9 gm/100 mL) was used to ensure uniformity of carbohydrate load, palatability, and voluntary intake among participants. Studies in children using serial magnetic resonance imaging have confirmed that 3 mL.kg^-1^ of such beverages return to baseline gastric volumes within an hour, making this volume a safe choice.^4^ No premedication was given. Anxiety was assessed using modified Yale Preoperative Anxiety Scale (mYPAS). Thirst was assessed using a binary scale. All patients underwent standardized inhalational induction with sevoflurane and nitrous oxide. Compliance during induction was evaluated using Induction Compliance Checklist (ICC). After induction, intravenous fentanyl (1–2 µg.kg^-1^) was administered, followed by laryngeal mask insertion. Blood glucose levels were measured at induction ‒ Fasting Blood Glucose (FBG) and before emergence. Maintenance anesthesia was with sevoflurane in oxygen/nitrous oxide and Ringer lactate fluid infusion. Upon completion, sevoflurane was discontinued, and 100% oxygen was administered. Intravenous ondansetron (0.1 mg.kg^-1^) and paracetamol (15 mg.kg^-1^) were given. After awake removal of the airway device, patients were transferred to the Post-Anesthesia Care Unit (PACU). ED was evaluated every 5 minutes for 30 minutes using the Pediatric Anesthesia Emergence Delirium (PAED) scale. ED was defined as PAED score > 10.^5^ Pain was assessed using Face, Legs, Activity, Cry, Consolability (FLACC) scale. Vital signs were continuously monitored. The primary outcome of the study was incidence of ED in first 30 min in PACU. The secondary outcomes were a) Association of fasting and emergence blood glucose levels with ED and b) Correlation between preoperative fasting duration and FBG level.

SPSS Version 23.0 was used to analyse the data. The normality of data was checked using Shapiro-Wilk test. Independent *t*-tests and Mann-Whitney *U*-tests were used as appropriate. Categorical variables were compared using Chi-Square or Fisher’s exact test. Spearman’s correlation assessed relationships between fasting duration and FBG values. Significance was set at p < 0.05. Based on prior ED incidence (∼44.4%) in children undergoing ocular surgery,^6^ a sample size of 39 per group (allowing for 10% dropout) was calculated to detect a 30% reduction in ED with 80% power (α = 0.05).

Seventy-eight children were randomized (39 per group). The Table 1 depicts the patients’ demographic and clinical characteristics. Eleven (28.2%) patients in group CD and 9 (23.1%) patients in group C exhibited postoperative ED (p = 0.60, OR = 1.31; 95% CI: 0.47, 3.63). Children who developed ED had lower mean FBG and emergence glucose values compared to those without ED, but these differences were not statistically significant (p = 0.29 and 0.10, respectively). No significant correlation was found between clear fluid fasting duration and FBG (Spearman’s p = 0.16, p = 0.17). A multivariable logistic regression analysis was performed to adjust for potential confounders including age, fasting duration, preoperative anxiety, quality of induction and duration of anesthesia. In the adjusted logistic regression model, clear fluid fasting duration was independently associated with a higher likelihood of ED (OR = 1.87, 95% CI: 1.10–3.18, p = 0.02). The other factors were not statistically significant predictors in this model.

**Table 1:** Demographic and clinical characteristics of the studied patients.

	Carbohydrate drink group (n = 39)	Control group (n = 39)	p-value
Age (years)	3.87 ± 1.45	3.73 ± 1.63	0.55
Gender: Male/Female	19 (48.7) / 20 (51.3)	15 (38.5) / 24 (61.5)	0.36
Weight (kg)	15.88 ± 3.95	15.46 ± 4.13	0.80
Type of admission: Out-patient / In-patient	23 (59.0) / 16 (41.0)	28 (71.8) / 11 (28.2)	0.23
Fasting for clear fluids (hours)	3.09 ± 0.83	5.23 ± 3.04	0.004
ASA Status I	39 (100)	39 (100)	1.00
Duration of procedure (minutes)	58.74 ± 4.55	58.41 ± 7.04	0.44
Discharge from PACU readiness (minutes)	60.00 ± 15.73	59.49 ± 14.68	0.89
Preoperative thirst	25 (64)	26 (67)	0.81
Preop mYPAS score	26.67 (23.33‒41.67)	28.33 (23.33‒41.67)	0.48
ICC score	0 (0–1)	1 (0–2.5)	0.31
FLACC score	0 (0–2)	0 (0–3)	0.39
Fasting blood glucose (mg.dL^-1^)	80 (76–87)	83 (79–86.5)	0.3
Blood glucose at emergence (mg.dL^-1^)	82 (77.5–86)	79 (76–84)	0.21

PACU, Postanesthesia Care Unit; ASA, American Society of Anesthesiologists; mYPAS, modified Yale Preoperative Anxiety Scale; ICC, Induction Compliance checklist; FLACC score, Face, legs, activity, cry and consolability score.

^†^ Values are expressed as numbers (percentage), mean ± SD or median (IQR).

^§^ Independent *t*-tests and Mann-Whitney *U* tests were used as appropriate.

Our study found no difference in the incidence of ED between the intervention and control group. Several factors may explain this finding. First, the volume of carbohydrate beverage administered may have been insufficient to produce a measurable physiological or behavioural effect. Previous studies using larger volumes (6‒15 mL.kg^-1^) of preoperative oral fluids have reported reduction in preop irritability among children.^7^^,^^8^ Second, ED is a multifactorial phenomenon ‒ despite standardized anesthesia protocols, individual variations in baseline anxiety levels, drug dosages, anesthetic agents, anesthesia depth, emergence conditions or postoperative pain may have influenced outcomes. However, the absence of significant differences in FLACC scores between children with and without ED suggests that pain was not a major contributing factor to the observed emergence behaviors. Third, the study population ‒ healthy children undergoing short-duration elective surgery ‒ might inherently have a lower baseline ED risk, reducing the likelihood of detecting a treatment effect. Finally, only the outcome assessors were blinded-introducing a risk of potential performance bias.

Glucose plays a key role in supporting neurotransmitter activity and cognitive function, and fluctuations in glycemia have been associated with agitation and impaired emergence behaviors.^2^ By maintaining euglycemia, carbohydrate loading may offer partial neurobehavioral protection during emergence, addressing one component of the complex ED pathophysiology. In our study, children who developed ED had lower mean fasting and emergence glucose levels, but the differences were not statistically significant. These findings align with prior studies that suggest a potential but not definitive link between perioperative hypoglycemia and behavioral disturbances.^8^ The limited sample size along with the modest glycemic differences observed may have been insufficient to elicit a clinically meaningful effect.

The lack of a statistically significant correlation between preoperative fasting duration and FBG levels indicates considerable interindividual variability owing to factors such as patients’ age, nutritional status, carbohydrate type and content of foods and drinks ingested in preoperative period, baseline metabolic rate and hormonal regulation modulating perioperative glycemic responses independently of fasting duration.

Despite standardized preoperative instructions, a variability in actual fasting durations was observed among participants in both groups. This discrepancy is likely attributable to practical scheduling constraints, operating room logistics, and variability in surgery start times**-**common challenges in pediatric surgical settings. While we aimed to adhere strictly to the fasting protocol, such deviations reflect the real-world clinical environment**,** where delays or early scheduling changes can extend or shorten fasting periods unpredictably. Longer fasting duration was found to be independently associated with higher odds of ED.

In summary, oral carbohydrate preloading did not significantly reduce the incidence of ED in this population. Further studies are warranted to explore optimal carbohydrate composition, volume, and timing, and potentially examine additional outcomes such as parental satisfaction, perioperative stress markers, and neurocognitive recovery.

Ethical approval, CTRI registration and consent to participate: Ethical approval for the study was obtained from Intramural Institutional Ethics Committee (NK/7926/MD/547) on 25 November 2021. The study was prospectively registered with Clinical Trial Registry of India (CTRI/2021/12/038688). [Registered on: 16/12/2021]. Informed written consent from the parents was obtained for inclusion in the study.

## Funding/Grant/Support

None.

The contents of the article have not been published elsewhere and the paper is not being submitted elsewhere. The manuscript has been read and approved by all co-authors. We hereby transfer, assign, or otherwise convey all copyright ownership, including any and all rights incidental thereto, exclusively to the journal, in the event that such work is published by the journal.

## Data availability statement

The datasets generated and/or analyzed during the current study are available from the corresponding author upon reasonable request.

## Conflicts of interest

The authors declare no conflicts of interest.

## Appendix Supplementary materials

Image, application 1

## References

[1] Smith M.D., McCall J., Plank L., Herbison G.P., Soop M., Nygren J. (2014). Perioperative oral carbohydrate therapy in elective surgery. Cochrane Database Syst Rev.

[2] Kanaya A., Nakahira J., Kanda N., Fujiwara Y., Niimi Y. (2019). The impact of intraoperative blood glucose on postoperative delirium. Anesth Analg.

[3] Oh A.R., Lee D.Y., Lee S. (2024). Association between Preoperative Glucose Dysregulation and Delirium after Non-Cardiac Surgery. J Clin Med.

[4] Andersson H., Frykholm P., Gilja O.H., Stenqvist O., Olsson C. (2018). Gastric emptying of carbohydrate drinks in children: a randomized controlled MRI study. Br J Anaesth.

[5] Aouad M.T., Yazbeck-Karam V.G., Nasr V.G., El-Khatib M.F., Kanazi G.E., Bleik JH. (2007). A single dose of propofol at the end of surgery for the prevention of emergence agitation in children undergoing strabismus surgery during sevoflurane anesthesia. Anesthesiology.

[6] Lin Y., Shen W., Liu Y. (2018). Visual preconditioning reduces emergence delirium in children undergoing ophthalmic surgery: a randomized controlled trial. Br J Anesth.

[7] Splinter W.M., Stewart J.A., Muir JG. (1990). Large volumes of apple juice preoperatively do not affect gastric pH and volume in children. Can J Anesth.

[8] Zamora C.C., Peralta L.A.C., Navaocampo AA. (2005). Randomized trial comparing overnight preoperative fasting period vs oral administration of apple juice at 06:00–06:30 AM in pediatric orthopaedic surgical patients. Pediatr Anesth.

