# Supplementary material for: Effect of preoperative clear carbohydrate beverage on emergence delirium in children – a randomized controlled trial
Source: Braz J Anesthesiol. 2025 Nov 3;76(1):844698. doi: 10.1016/j.bjane.2025.844698 (PMC12719025; doi:10.1016/j.bjane.2025.844698)
Supplement: Supplementary file 1 [file mmc1.docx]

**BJAN-D-25-00400_Supplementary Material**

**Supplementary Material**

**Table** PAED scores at various time points in PACU.

| **Time point** | **Carbohydrate drink group (n = 39)** | **Control group**  **(n = 39)** | **p-value** |
| --- | --- | --- | --- |
| PACU – 0 minutes | 7 (6–11) | 7 (6–10) | 0.78 |
| PACU – 5 minutes | 5 (4–6.5) | 5 (4–7) | 0.87 |
| PACU – 10 minutes | 4 (3–4) | 4 (3–4) | 0.26 |
| PACU – 15 minutes | 3 (0–3) | 3 (0–3) | 0.79 |
| PACU – 20 minutes | 0 (0–0) | 0 (0–0) | – |
| PACU – 25 minutes | 0 (0–0) | 0 (0–0) | – |
| PACU – 30 minutes | 0 (0–0) | 0 (0–0) | – |

† Values are presented as median (IQR).

‡ PACU, Post Anesthesia Care Unit.

§ Mann-Whitney *U* test was used to obtain p-values.

**Table** Multivariable Logistic Regression Model adjusting for potential confounders.

| **Variable** | **Adjusted OR** | **95% CI** | **p-value** |
| --- | --- | --- | --- |
| Age (years) | 0.88 | 0.60–1.28 | 0.50 |
| Fasting duration for clear fluids (hours) | 1.87 | 1.10–3.18 | **0.022** |
| Preoperative anxiety score | 0.98 | 0.94–1.1 | 0.28 |
| Quality of induction | 0.83 | 0.59–1.16 | 0.28 |
| Duration of anesthesia (hours) | 0.89 | 0.78–1.02 | 0.1 |
